# The clinician’s perspective on the 21-gene assay in early breast cancer

**DOI:** 10.18632/oncotarget.28148

**Published:** 2021-12-21

**Authors:** Francesco Cognetti, Giuseppe Naso

**Affiliations:** ^1^Department of Clinical and Molecular Medicine, University La Sapienza, Rome, Italy

**Keywords:** Oncotype DX test, recurrence score results, multigene assays, genomic tests, personalized medicine

## Abstract

Most patients with early HR+ and HER2- breast cancer receive a hormone therapy; the clinical question still open is how to identify patients who can really benefit from adjuvant chemotherapy. The accurate identification of these patients is essential to avoid an over-treatment, increasing the risk of an unnecessary toxicity; on the contrary, the omission of chemotherapy can deprive high risk patients of a potential life-saving treatment (under-treatment). Several multigene assays (MGAs), assessing the risk of relapse according to the biological characteristics of the tumor, have been developed. To date, the 21-gene assay (Oncotype DX Breast Recurrence Score^®^) is the only test developed and validated to be actionable, i.e., able to predict the benefit of adjuvant chemotherapy. The different available tests can be classified according to their clinical utility based on their prognostic and predictive value. A prognostic test gives information about the outcome of the disease, regardless of the administered therapy. When the aim of the test is to drive the treatment decisions, the predictive component, and therefore the ability to accurately identify which patients could benefit from chemotherapy, is essential. This review summarizes the clinical evidences of the Oncotype DX^®^ test supporting its clinical utility.

## INTRODUCTION

Most patients with early HR+ and HER2− breast cancer receive a hormone therapy; the clinical question still open is how to identify patients who can really benefit from adjuvant chemotherapy. The accurate identification of these patients is essential to avoid an over-treatment, increasing the risk of an unnecessary toxicity; on the contrary, the omission of chemotherapy can deprive high risk patients of a potential life-saving treatment (under-treatment). This review analyzes the main characteristics of the genomic tests developed to help oncologists to improve the patient’s prognosis, with a special focus on Oncotype DX^®^.

## THE TREATMENT OF THE EARLY BREAST CANCER (EBC)

### Classification and epidemiology

The breast cancer represents 24% of all tumors and it is globally responsible for 15% of cancer death. Breast cancer is the most frequent cancer in women and the first cause of death for cancer in women. In Italy [1] 53.000 new cases of breast cancer have been estimated in 2019. The breast cancer is the most diagnosed cancer in women (30% of malignant tumours), with a different rate depending on age: 40% in patients aged 0–50 years; 35% in patients aged 50–69 years and 22% in patients aged >70 years.

### The personalized medicine in early breast cancer

Estrogen Receptors (ERs) and/or Progesterone Receptors (PgRs) are present on the surface of the neoplastic cells in about 75% of all breast cancers. These tumors, called luminal tumors due to the fact that they seem to originate mainly from the luminal cells of the galactophor ducts and lobules, are all theoretically hormone-responsive at any stage of disease and they are mainly treated with hormone therapy (SERM, LHRH Analogues, Aromatase Inhibitors (AIs), SERD). In case of metastatic disease, a m-TOR inhibitor (Everolimus), an inhibitor of the catalytic domain of Phosphatidyl Inositol 3-Chinase (PI3KCa) or a CDK4/6 (cyclin-dependent kinase 4 and 6) inhibitor may be added to the hormone therapy, if the patient’s condition allows it. In patients undergoing surgery for early stage hormone-responsive breast cancers (local or locally advanced for involvement of ipsilateral axillary lymph nodes), the standard therapeutic approach for the prevention of local disease recurrence and metastases mainly consist of hormone therapy (HT: LHRH-analogues, SERM, AIs). The main challenge of oncologists is to decide when to consider hormone therapy alone or when to add chemotherapy (CT).

### Risk evaluation

The risk of distant relapses is continuous, especially for ER + breast cancers, which account for almost half of metastatic cases [2]. To date, it is not clear whether adjuvant CT can effectively prevent these recurrences. An overview performed by the Early Breast Cancer Trialists’ Collaborative Group (EBCTCG) in 2012, showed a limited benefit from CT (about 10% of patients) and, mostly, the inability to identify a subgroup of patients who could significantly benefit from CT, based on clinical and pathological characteristics only [3]. The choice of the adjuvant CT is therefore particularly complex and based on histological, pathological and immunohistochemical characteristics of the tumour, as well as on the patient’s condition.

The main prognostic parameters used to evaluate the risk of metastasis, and consequently the most suitable therapy, are: age (<35 years: worst prognosis), tumour size, histological grade and type (G), presence of axillary lymph nodes metastases, lympho-vascular invasion, hormonal receptor status (HR), human epidermal growth factor 2 (HER2) receptor status, proliferative activity (evaluated through the molecular marker Ki-67) and gene expression profiles [4].

All these parameters allow oncologists to establish the prognosis of the patient with a good approximation; consequently, the therapeutic choice is based on the extrapolation that CT combined with hormone therapy is required in patients with a worse prognosis. High proliferative ER+/HER2− tumours are considered chemo-sensitive; on the contrary, low proliferative ER+/HER2− tumours hormone-sensitive [5].

However, traditional parameters are not able to establish the proliferative activity of the tumour in a reproducible and reliable manner [4]. For example, the evaluation of the proliferative activity with the Ki-67 labeling index, a nuclear antigen expressed during cell proliferation phases (G1, S, G2, M) but not by quiescent cells, is not reproducible due to the lack of standardized procedures for reading and interpreting results [6]. Relying only on clinical parameters, oncologists have some tools able to predict the prognosis of the disease and the benefit coming from hormone therapy (presence of ER), but they have no predictive data of the possible benefit from adding CT. Moreover, the interpretation of the clinical parameters is different among oncologists, so there is a lack of homogeneity of the adjuvant therapy for women with breast cancer after surgery. Finally, the use of unnecessary CT is associated with considerable burden for the patient who do not derive any clinical benefit.

### Critical aspects of the adjuvant therapy

Chemotherapy can cause a temporary or permanent moderate-severe disability, which has a negative impact on the quality of life and professional career of patients and family members [7]. Patients treated with CT tend to be absent from work longer (9.5 months with CT vs. 5.4 months without CT) and are often forced to leave their professional career (21% with CT vs. 14% without CT). Similarly, 52% of family members of patients receiving CT are absent from work for providing care [8].

The importance of carefully assessing the toxicity of the CT compared to its potential benefits is strictly linked to the possible long-term side effects, arising during the treatment period and generally resolving within a few months, and “late-chronic” side effects, occurring many years after the end of adjuvant CT and often lasting for several years [9, 10].

**Figure 1:**
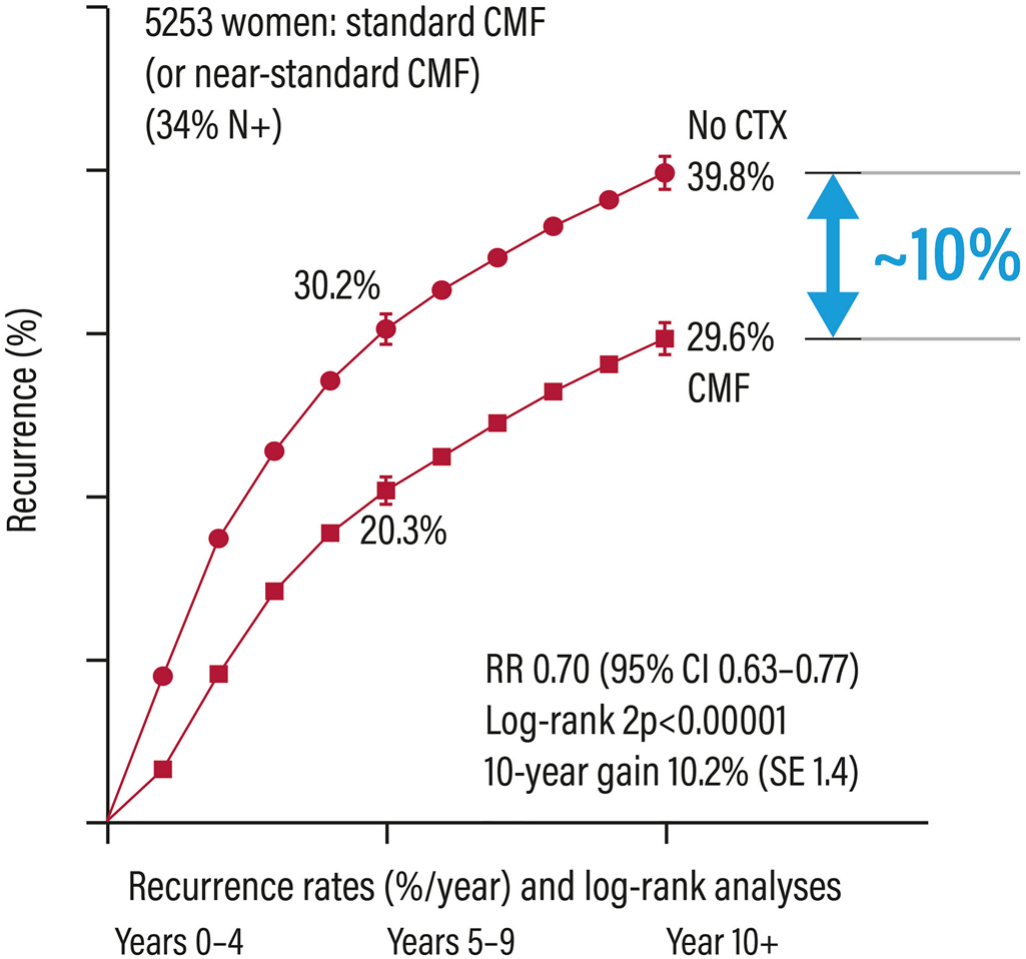
Relapse rates in patients with early breast cancer treated or not with adjuvant chemotherapy. Abbreviations: Cl: confidence interval; CMF: cyclophosphamide/methotrexate/5-fluorouracil; CTX: chemotherapy; RR: risk ratio.

Only 10% of patients with early ER+ breast cancer, which account for about 75% of breast cancers [4], clearly benefit from CT (Figure 1) [3]. It is therefore important to have decision-making tools driving the treatment choice, in order to avoid the CT-associated toxicity. The analysis of the tumour gene profile is essential in patients with ER+ cancers for better defining the prognosis and, when possible, being predictive of treatment response for driving the therapeutic choice related to the use of CT [5].

Genomic tests, developed and marketed in recent years could be a possible way to meet these objectives [4].

## MULTIGENE ASSAYS SUPPORTING THE CLINICAL DECISIONS

### The MGAs approved in Italy

Due to the clinical and molecular heterogeneity of breast cancer, several genomic tests have been developed to help oncologists to improve the patient’s prognosis (Oncotype DX, EndoPredict, Prosigna, MammaPrint) and to predict chemotherapy benefit in order to choose the most appropriate adjuvant therapy (Oncotype DX) [4].

In Italy, all 4 genomic tests are available for early breast cancers. The tests are not equivalent: in fact, they have been designed to evaluate the different gene profile and to address different questions (Table 1).

**Table 1:** Breast cancer genomic signature platforms

Oncotype DX^®^, EndoPredict^®^	MammaPrint^®^	Prosigna™
RT-PCR gene expression	RNA gene microarray	Hybridization with gene specific labels
Quantitative (continuous)	Qualitative (binary)	Quantitative (continuous)
Gene expression levels of relatively small number of genes	Gene expression levels of large number of genes	Gene expression levels of relatively large number of genes
High precision	Modest precision	Moderate to high precision

### Differences and characteristics of the MGAs approved in Italy: prognostic and predictive value

Table 1 shows the main characteristics of the 4 MGAs. Due to the different gene panels, genomic tests cannot be considered similar. The tests also differ in their purposes (prognostic and/or predictive value) and consequently in the different validation studies.

The possibility to provide an estimate of the relapse risk (prognostic value) compared to the benefit from CT (predictive value) is undoubtedly one of the most important and distinctive aspects. A prognostic test provides information on the natural history of the disease and on the risk an event appears (e.g., distant relapse). Therefore, prognostic tests can suggest the need for adjuvant therapy, but do not give information about the probability of response to a specific treatment. A genomic prognostic test is generally validated by a retrospective analysis of biopsy samples collected in randomised controlled clinical trials or observational trials performed on untreated patients (as in case of MammaPrint) or patients treated with hormone therapy alone (Endopredict, Prosigna, and the Oncotype DX test) [11]. The relationship between the test and the clinical outcome of the patient treated with hormone therapy will establish the prognostic value of the biomarker and complement traditional clinical pathological factors to refine prognosis.

A biomarker is considered “predictive” if it is associated to a clinical outcome in a strictly treatment-dependent manner and it is generally validated in prospective studies. Thus, a genomic test for early breast cancer is truly predictive when a statistically significant correlation among biomarker, the treatment group (es. CT vs. no CT) and the patient’s outcome is demonstrated [12].

Today, the Oncotype DX test is the only test considered predictive, supported by evidence from clinical studies designed to assess its clinical utility in guiding clinicians in their therapeutic choice. Clinical evidence shows that, overall, patients with a high value of the Recurrence Score results (RS 26–100) benefit from CT, while high values of other genomic tests, such as Prosigna or EndoPredict, reflect a high risk for recurrence without information on benefit from treatment [13]. Similarly, the MINDACT trial did not provide definitive information on the benefit of adjuvant CT for patients with high MammaPrint results [14].

In our clinical practice our main question is to identify the few patients who could effectively benefit from chemotherapy; hence we focus this review on the predictive test for chemotherapy, the Oncotype DX test.

## THE ONCOTYPE DX TEST

**Figure 2:**
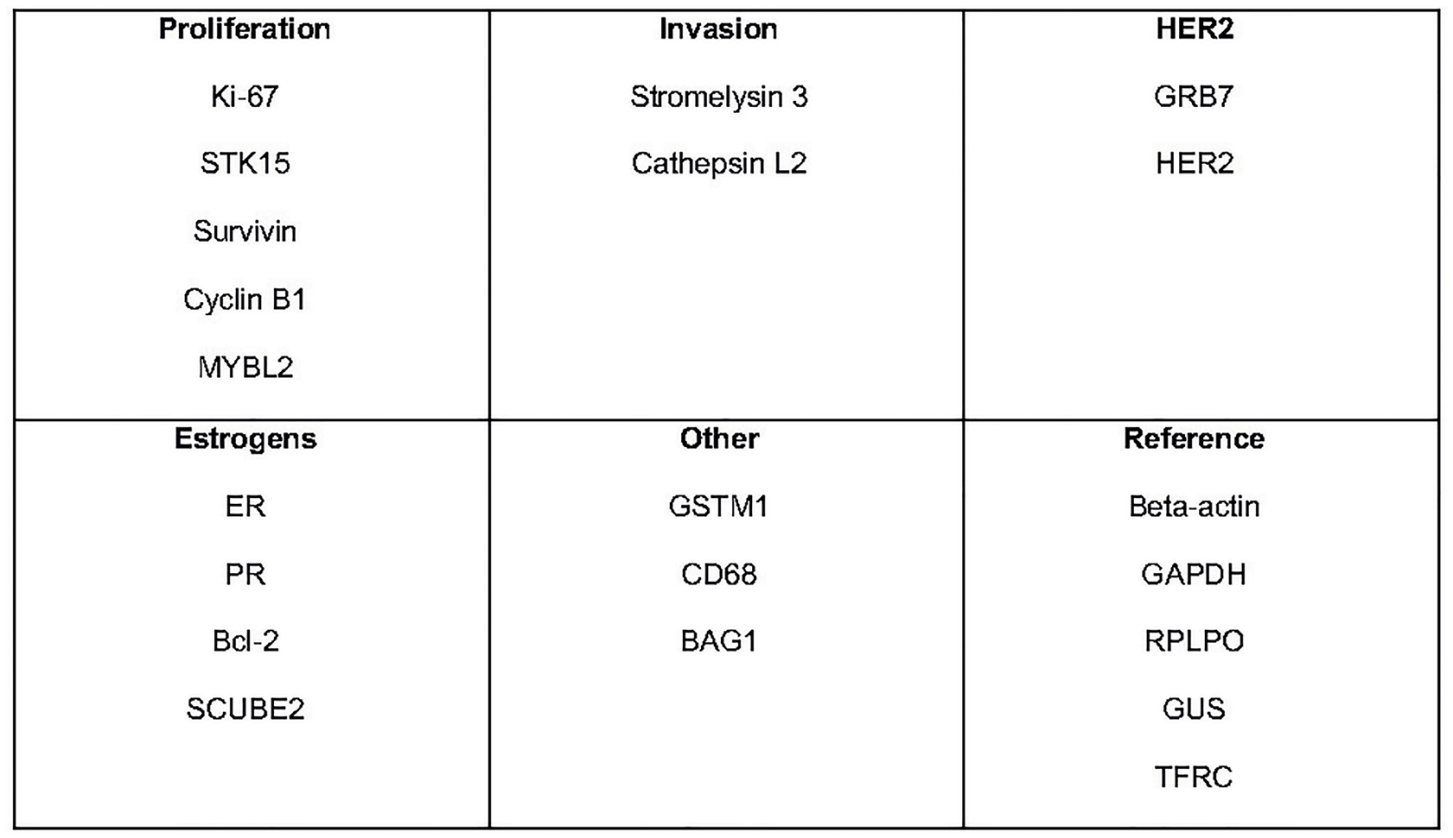
Specific cancer genes and reference cancer genes analized with the Oncotype DX text. Abbreviations: STK15: Serine/Treonine kinasi 15; MYBL2: Myb-related protein B; GRB7: Growth factor receptor-bound protein 7; HER2: human epidermal growth factor receptor 2; ER: Estrogen receptor; PR: Progesterone receptor; Bcl-2: B-cell lymphoma 2; SCUBE2: Peptide signal, CUB damain, epidermal growth factor receptor-like 2; GSTM1: Glutathione S-transferase mu 1; CD68: Cluster differentiation 68; BAG1: Bcl-2 associated athanogene-1; GAPDH: Glyceraldehyde-3-Phosphate Dehydrogenase; RPLPO: Large Ribosomal Protein; GUS: glucuronidase; TFRC: Transferrine receptor.

The Oncotype DX test is a molecular test based on the qRT-PCR technology aimed to evaluate the expression of 21 genes (Figure 2) on a surgical or core biopsy sample of neoplastic breast tissue. The analysis is indicated in patients with early HR+, HER2− N0 or N1 (1–3 lymph nodes) breast cancer and can provide the likelihood of long-term tumour relapse (prognostic component), as well as the possible response to adjuvant CT (predictive component).

### Standard and quality control

The tumour samples are sent to a centralized laboratory in a specially prepared container, enclosed in a transport kit. The test is performed in the United States on a single platform according to the required quality standards (Clinical Laboratory Improvement Amendments – CLIA – Certification, ISO 15189). The Oncotype DX analysis methodology and the strong quality standard imposed lead to a highly standardized and validated test.

### Analysis

The procedure in based on the extraction of RNA from formalin-fixed and paraffin-embedded tumour tissue (FFPE) treated with Deoxyribonuclease I (DNase I). The total amount of RNA is then measured, after verifying the absence of a DNA contamination. Once RT-PCR is performed, the expression of each of the 16 selected genes is evaluated in triplicate and then normalized to the expression of the 5 reference genes (Figure 2). This normalization minimizes any sources of pre-analytical variability [15].

**Figure 3:**
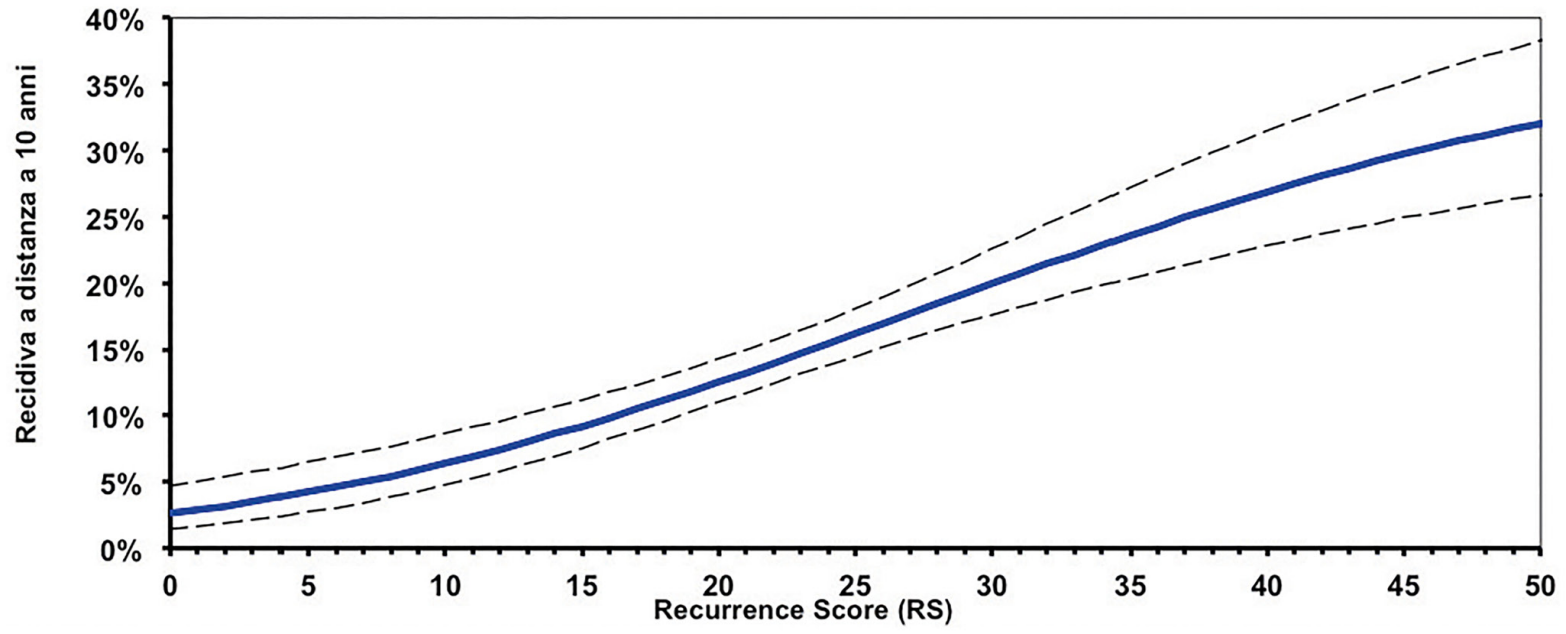
Continuous correlation between Recurrence Score (RS) results and risk of relapse.

The results of this analysis are then combined, using an algorithm, into a single score known as Recurrence Score (RS) result, expressed on a continuous scale from 0 to 100. The RS results provides for the patients the probability of CT benefit in addition to hormone therapy over the next 9 years, with a 95% confidence interval (CI). The quantitative nature of the PCR allows a continuous score to be obtained, which differs from the binary result (only low or high) provided by other tests (Figure 3).

## VALIDATION STUDIES

### Analytical validation

An analytical validation process was put in place for the Oncotype DX test too, in order to ensure that the laboratory procedures implemented to calculate the RS results were accurate, precise and reproducible.

The validation of the test was performed for the single genes used in the test as well as for the results obtained with the RS results. The studies performed confirmed the reproducibility, the accuracy and the precision of the test, and showed that the RS results are expressed in a more dynamic range than other analytical methods. In addition, sensitivity, specificity, detection and quantification limits, amplification efficiency and test success rates were demonstrated, confirming that the test performance (in terms of uniformity in amplification efficiency for different genes and linearity) was maintained over a wide range [15].

### Clinical validation of the prognostic value

The clinical validation studies of Oncotype DX test were mostly performed in 2 different populations node-negative patients and node-positive HR+, HER2− early breast cancer patients. Table 2 summarizes the studies which evaluated the prognostic and predictive value of Oncotype DX test.

**Table 2:** Clinical studies and real-world evidence with the Oncotype DX test

Reference	Study type	N patients	Treatment	Nodal status	Prognostic value	Predictive value
**Paik et al. 2004 [16]**	Prospective-Retrospective	668	adjuvant ET	pN0: 100%	Yes	–
**Gianni et al. 2005 [17]**	Retrospective	89	neoadjuvant CT	cN0: 16% cN+: 84%	–	Yes
**Paik et al. 2006 [18]**	Prospective- Retrospective	651	randomized adjuvant ET or ET + CT	pN0: 100%	Yes	Yes
**Chang et al. 2008 [19]**	Prospective-Retrospective	80	neoadjuvant CT	cN0: 90% cN+: 10%	–	Yes
**Dowsett et al. 2010 [20]**	Prospective- Retrospective	1.231	adjuvant ET	pN0: 71% pN+: 25%	Yes	–
**Albain et al. 2010 [21]**	Prospective- Retrospective	367	randomized adjuvant ET or ET + CT	pN+: 100%	Yes	Yes
**Petkov et al. 2016 [22]**	Prospective Registry	25.714	adjuvant ET or ET + CT	pN0: 82% pN+: 18%	Yes	–
**Roberts et al. 2017 [23]**	Prospective Registry	6.768	adjuvant ET or ET + CT	pN+: 100%	Yes	–
**Stemmer et al. 2017 [24]**	Prospective Registry	1.801	adjuvant ET or ET + CT	pN0: 100%	Yes	–
**Stemmer et al. 2019 [25]**	Prospective Registry	1.365	adjuvant ET or ET + CT	pN1 : 100%	Yes	–
**Nitz et al. 2017 [26]**	Prospective	2.642	adjuvant ET	pN0: 59% pN+: 41%	Yes	–
**Mamounas et al. 2018 [27]**	Prospective- Retrospective	1.065	adjuvant ET + CT	pN+: 100%	Yes	–
**Sparano et al. 2018 [28]** **Sparano et al. 2019 [29]**	Prospective randomised	10.273	randomized adjuvant ET or ET + CT	pN0: 100%	Yes	Yes

Abbreviations: CT: chemotherapy; ET: endocrinotherapy.

The first study [16] validating RS results and the prognostic value of the Oncotype DX test (probability of recurrence at 10 years) was performed on biopsy samples of 668 patients with ER+ node-negative breast cancer, enrolled in the NSABP- B14 study originally conducted to evaluate the efficacy of 5 years of adjuvant treatment. The results showed that:

- RS results 0–17 was observed in 338 patients (51% low-risk patients) with a relapse rate of 6.8% at 10 years
- RS results 18–30 was observed in 149 patients (22% intermediate risk patients) with a relapse rate of 14.3% at 10 years
- RS results 31–100 was observed in 181 patients (27% high risk patients) with a relapse rate of 30.5% at 10 years.

**Figure 4:**
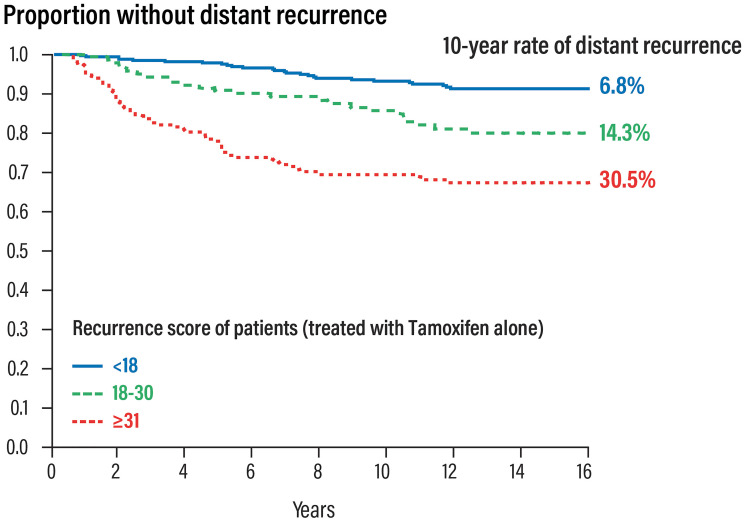
Prognostic validation of the Oncotype DX test in patients with early HR+ and nodes negative breast cancer.

The predicted relapse risk with the RS results was independent of other factors such as age, size and tumour grade (Figure 4).

The prognostic value of the Oncotype DX test was also validated in node-positive patients enrolled in the TransATAC trial [20, 30]. The RS results were significantly prognostic for the 9-years disease-free survival.

A further prospective study, WSG-plan B [26], enrolled node-negative patients at high risk and Patients node-positive patients with Recurrence Score results 0–11 Patients were treated with hormone therapy alone and showed that CT could be avoided. Patients with Recurrence Score results above 12 were randomized to different CT regimens: for this reason, they do not support the prognostic value in patients treated with hormone therapy alone.

### Clinical validation of the predictive value

The Oncotype DX test was the first and, so far the only, validated test able to identify chemo-sensitive patients who can obtain the highest benefit from adjuvant CT.

### NSABP-20 trial [18, 31]

Biopsy samples from the NSABP B-20 trial, which enrolled 651 patients with ER+ lymph node negative breast cancer, randomized to tamoxifen (227 patients) o tamoxifen plus CT (424 patients; CMF: cyclophosphamide/methotrexate/5-fluorouracil, or MF: methotrexate/5-fluorouracil) were used to assess the prognostic and predictive value of the Oncotype DX test. The trial was firstly analysed in order to demonstrate the predictive value of the Oncotype DX test [18]. The analysis has been revised to exclude HER2-positive patients initially included and to better define the cut-off value of the test [31].

**Figure 5:**
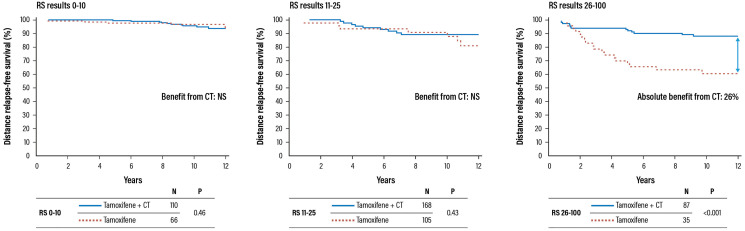
Validation trial of the Oncotype DX test for the identification of chemosensitive patients (HR+ and negative lymph notes). Abbreviations: CT: chemotherapy; HR: hazard ratio; NS: not significant; RS: recurrence score.

The study showed that patients with RS results 31–100 had a significant benefit from the addition of CT, with a 26% increase in the relapse-free survival (88% vs. 62%); on the contrary, patients with RS results 0–17 did not benefit at all from CT (Figure 5). The Recurrence Score result is a predictive factor for the benefit of CT (test for interaction between CT and RS groups statistically significant with *P* = 0.014).

### TAILORx trial [28, 32]

The predictive value of the Oncotype DX test to identify patients who could omit CT was further validated by the prospective randomized clinical trial TAILORx (Trial Assessing Individualized Options for Treatment) on 10,273 enrolled patients with ER+, HER2-negative N0 early breast cancer. The objective of this study was to evaluate the non-inferiority of hormone therapy versus chemo-hormone therapy in patients with RS results 11–25.

**Figure 6:**
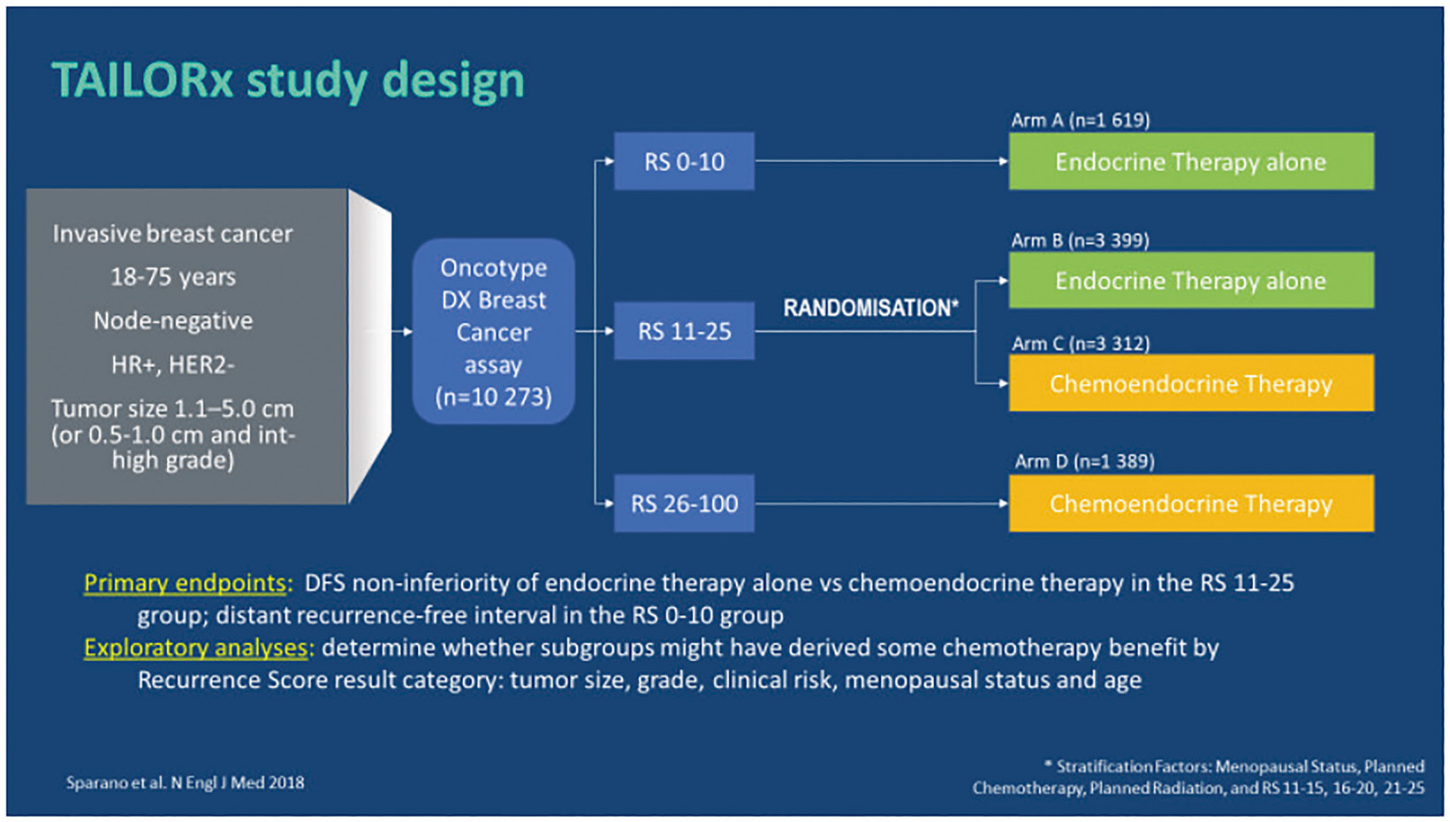
TAILORx: study design. Abbreviations: HER2: epidermal growth factor 2; HR: hormonal receptor; RS: recurrence score.

In the TAILORx trial (Figure 6) different ranges of RS results (0–10, 11–25, 26–100) were used in order to minimize the risk of under-treating the high-risk patients as well the randomized group patients [32]. Patients were stratified in 3 treatment groups [28]:

- RS results 0–10; *n* = 1,619; 17% of patients received hormone therapy alone;
- RS results 11–25; *n* = 6,711; 69% of patients were randomized to hormone therapy alone (*n* = 3,399) or chemo-hormone therapy (*n* = 3,312);
- RS results 26–100; *n* = 1,389; 14% of patients received chemo-hormone therapy (CT + hormone therapy).

The primary endpoint of the TAILORx trial was the distant disease-free survival (IDFS, invasive disease-free survival, defined as the absence of metastatic relapses according to primary carcinoma or death) in patients with RS results 11–25.

**Figure 7:**
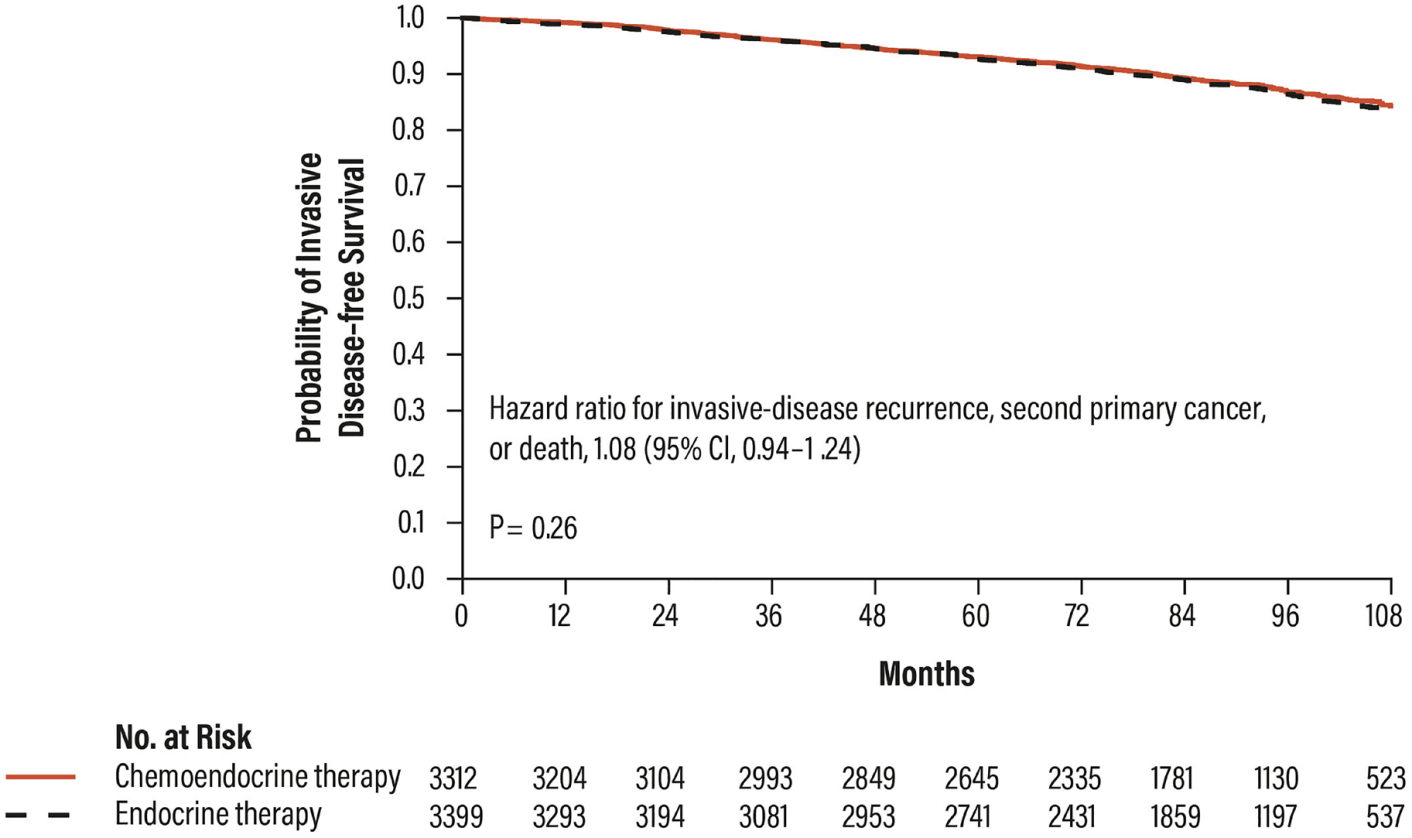
Distant disease-free survival in patients with RS results 11–25 in the TAILORx trial. Abbreviations: CI: confidence interval.

The results of the study demonstrated the non-inferiority of the hormone therapy respect to the chemo-hormone therapy (HR 1.08; CI 95% 0.94–1.24; *p* = 0.26), highlighting that patients with RS results 11–25 do not benefit from CT and can be treated with hormone therapy alone (Figure 7) [28].

**Figure 8:**
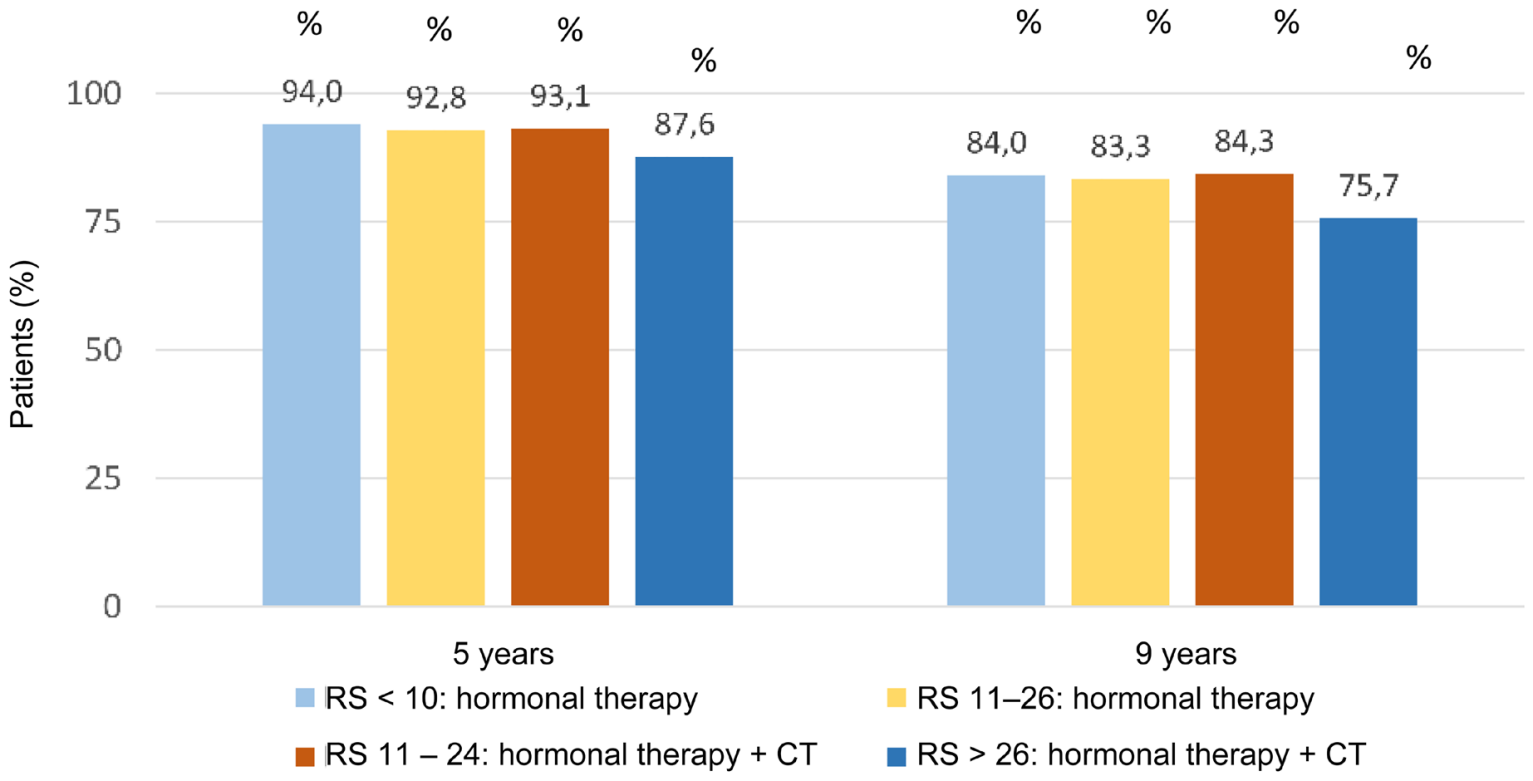
Invasive disease-free survival for RS results in TAILORx trail. Abbreviations: CT: chemotherapy; RS: recurrence score.

After 9 years, the 2 randomized groups of RS 11–25 patients had similar invasive disease-free survival rates: 83.3% in patients treated with hormone therapy alone and 84.3% in patients treated with chemo-hormone therapy. Similarly, the two treatment groups had similar distant relapse-free survival rates: 94.5% and 95% for hormone and chemo-hormone therapy, respectively. At 9 years, 84% of patients with RS results 0–10 were invasive disease-free and 96.8% distant relapse-free (Figure 8) [28].

Overall, these results show that the use of the Oncotype DX test allows to identify the small number of patients (about 20%) who can substantially benefit from CT, saving most of them (about 80%) from toxicity and chronic side effects related to CT.

**Figure 9:**
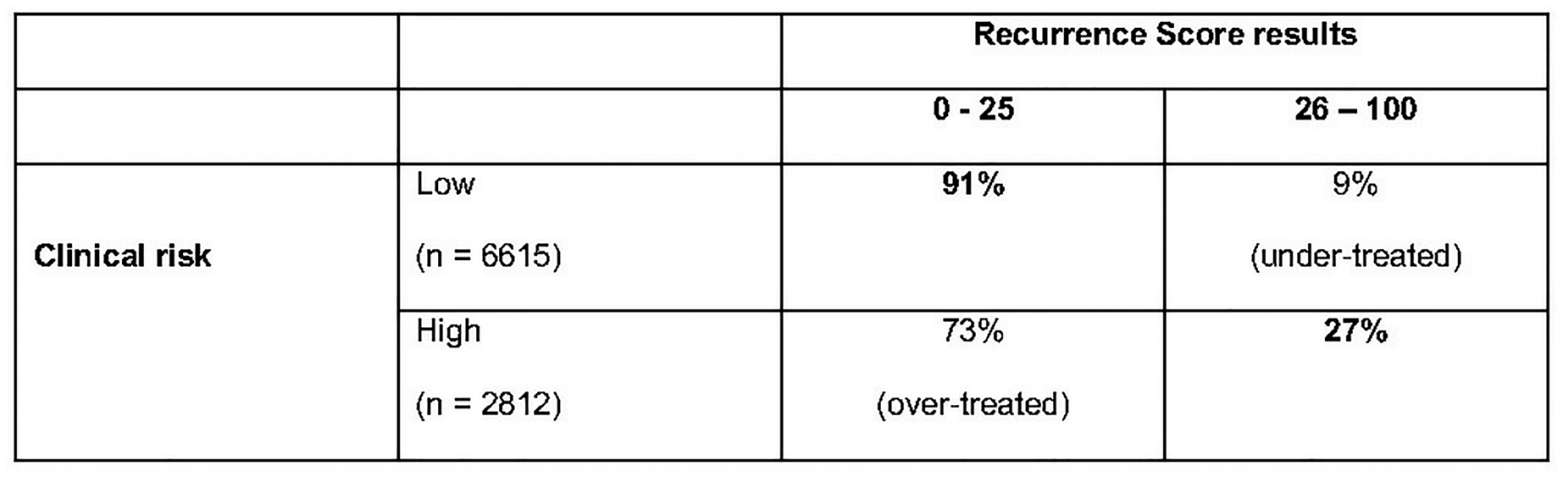
Proportion of high and low clinical risk patients with RS results 0–25 vs. 26–100 in TAILORx trail.

A study was performed in patients enrolled in the TAILORx study for whom both tumour size and histological grade data were available (97% of the total). 6615 out of the 9427 women with tumour size and histological grade data available had a low clinical risk (70.2%) and 2812 (29.8%) were classified as “high clinical risk”. A significant proportion (73%) of patients with RS results 0–25 had a high clinical risk and would have been over-treated if the decision on CT had been based on clinical risk parameters only (Figure 9). The RS was high (a score of 26 to 100) in 589 patients (9%) with low clinical risk. These patients would have been under-treated (Figure 9) [28].

The TAILORx trial shows that patients with RS results 0–25 do not benefit from CT. The NSABP 20 trials shows that patients with RS results 26–100 significantly benefit from CT.

### Secondary analysis of the TAILORx trial [29]

Sparano et al. performed secondary analyses of the TAILORx trial in order to establish whether the age and clinical risk, evaluated through modified Adjuvant!Online, a web-based tool that provides estimates of adjuvant therapy outcomes for individual patients, could add prognostic information to RS results and/or predictive information regarding the benefit of CT. While patients aged >50 years and RS results 11–25 did not benefit from CT, younger patients (age ≤50 years) started to show some benefit from CT with a RS result 16. The differences observed in this un-planned exploratory analysis, although not statistically significant, could have clinical relevance. This observation was further assessed showing that in 476 women with RS results 21–25, the absolute benefit of the CT in the low-clinical risk subgroup (6.4 ± 4.9%) was similar to that of the high-clinical risk subgroup (8.7 ± 6.2%). In the 886 women with RS results 16–20, an estimated benefit of CT was observed in the high-clinical risk subgroup (6.5 ± 4.9%) but not in the low-clinical risk subgroup (−0.2 ± 2.1%) [29].

**Figure 10:**
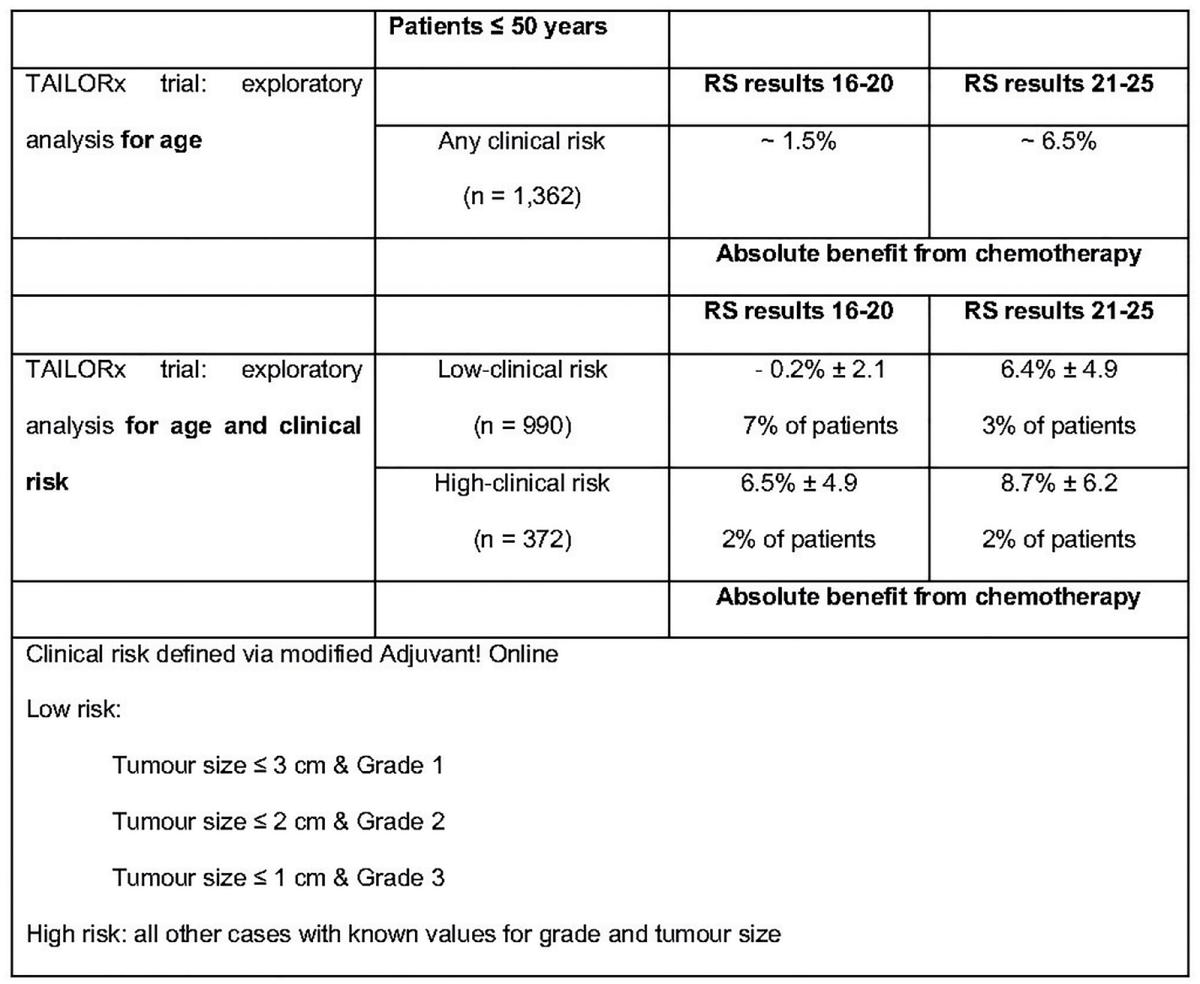
Exploratory un-planned analysis of TAILORx trail. Abbreviations: RS: recurrence score.

These results suggest that clinical-pathological risk factors, while by themselves are not able to predict a benefit from CT, they can help to optimize treatment decisions when used in combination with RS results, especially around cut-off values (Figure 10).

### SWOG 8814 trial [21]

In order to evaluate the prognostic and predictive value of the Oncotype DX test in HR+ and positive lymph nodes patients, biopsy samples of 367 patients randomized to tamoxifen (*n* = 148) and CAF + tamoxifen (*n* = 291) enrolled in the SWOG8814 trial were retrospectively analysed.

**Figure 11:**
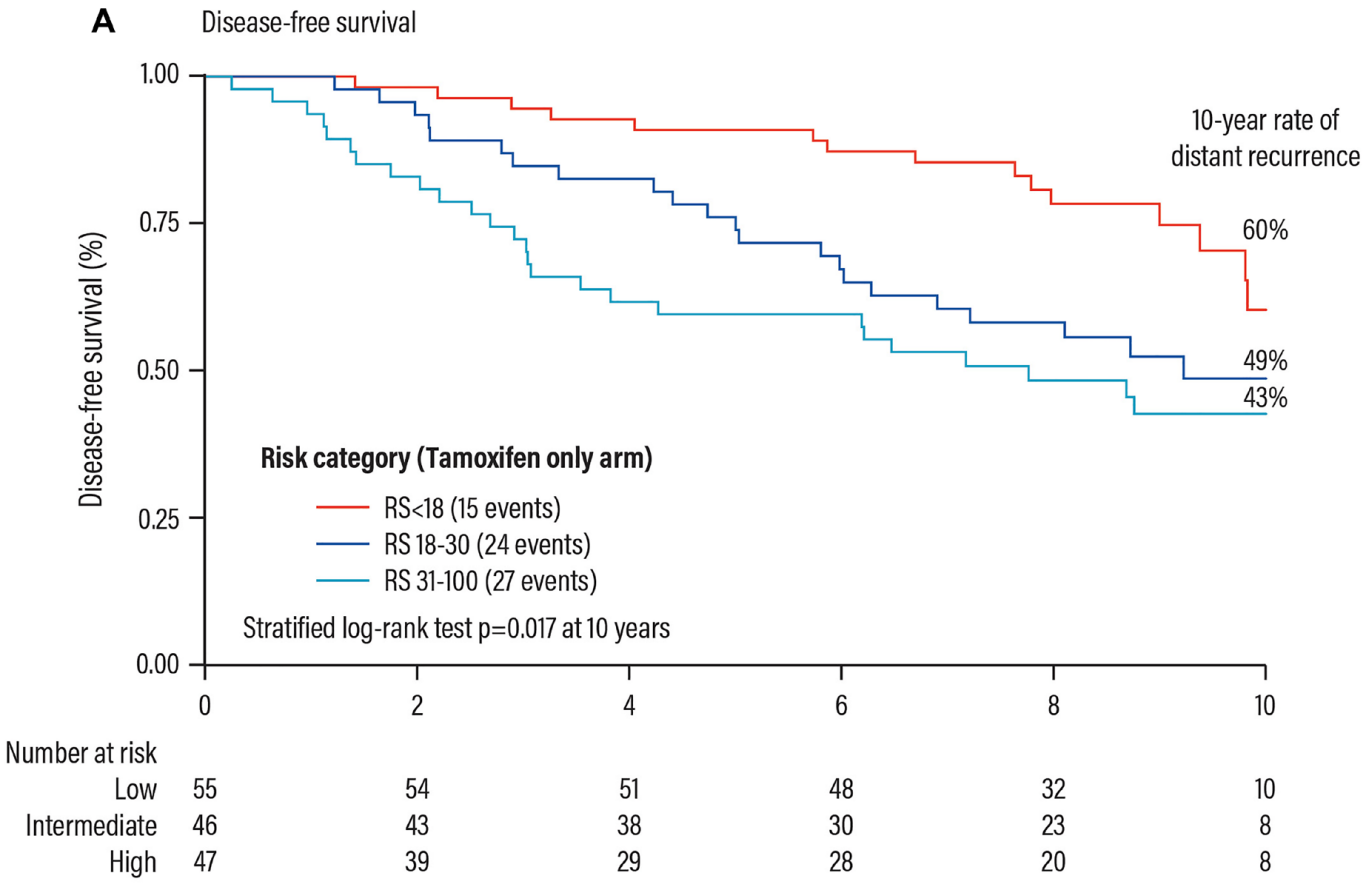
Prognostic validation of the Oncotype DX test in patients with HR+ early breast cancer. Abbreviations: RS: recurrence score.

**Figure 12:**
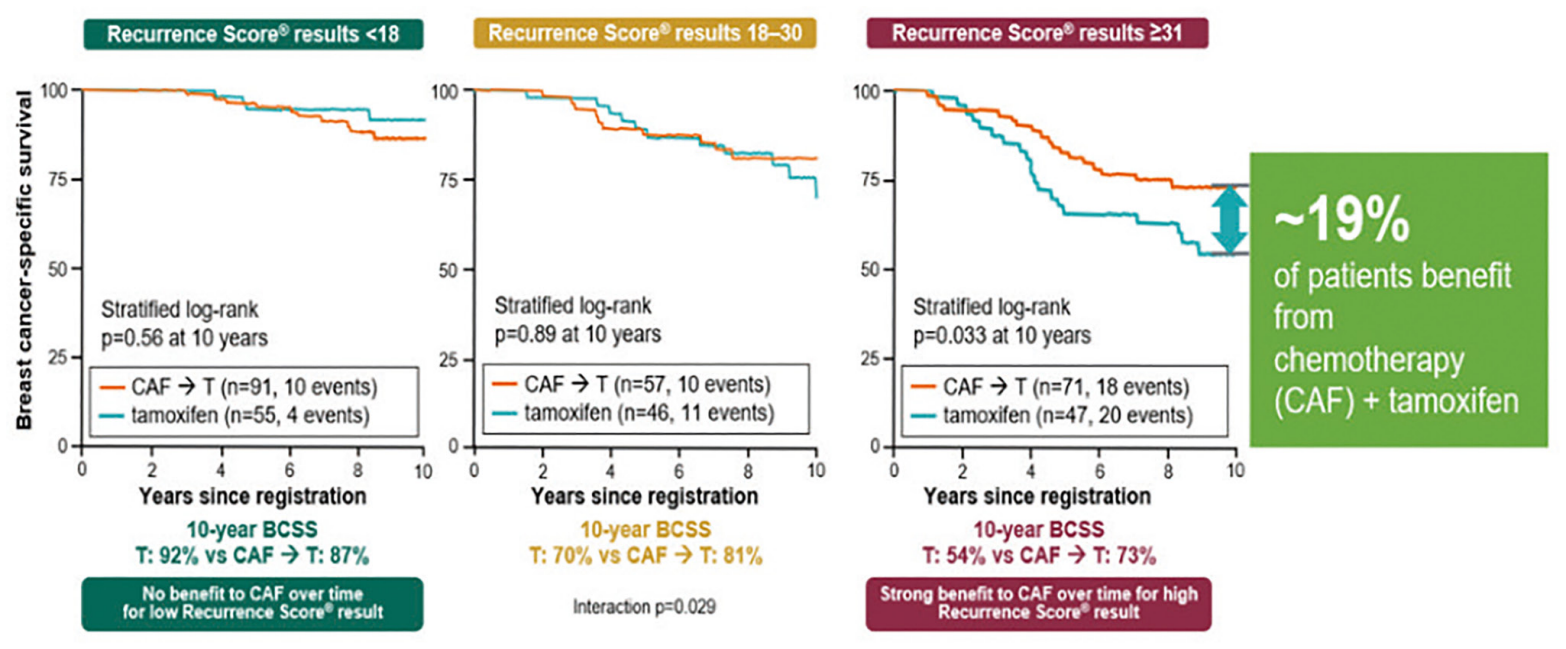
Validation trials of the Oncotype DX test for the identification of chemosensitive patients (HR+ and positive nodes) that could benefit from chemotherapy. Abbreviations: BCSS: breast cancer-specific survival; CAF: Cytoxan: Adriamycin and Fluorouracil.

The results showed that:

- The RS result is highly prognostic (progression-free survival) in the group of patients treated with tamoxifen only (Figure 11).
- The RS result is a strong predictor of the benefit from CT during the first 5 years (*p* = 0.029).
- Patients with RS results 31–100, derived a substantial 19% benefit from CT, patients with RS 0–17 did not derive benefit from CT in addition to hormonal therapy (Figure 12).

### RXPONDER (SWOG S1007 treatment for positive node, endocrine-responsive breast cancer)

The SWOG S1077 RXPONDER trial enrolled 5,015 patients with HR-positive, HER2-negative, stage 2–3, breast cancer involving 1–3 axillary lymph nodes and no distant metastasis. Eligible patients had RS of 0–25, indicating a low risk of recurrence. Patients were randomly assigned to receive chemotherapy plus standard adjuvant endocrine therapy (*n* = 2,547) or endocrine therapy alone (*n* = 2,536).

The primary endpoint is invasive disease-free survival (IDFS). The median follow-up of the current interim analysis is 5.1 years. Preliminary results were presented at the San Antonio Breast Cancer Symposium 2020 (ref. Oncologist. 2021 Feb; 26, Suppl 2: S11–S12). Compared with endocrine therapy alone, the addition of chemotherapy increased 5-year IDFS by 19% in the overall study population (91.0% vs. 92.4%; *p* = .026). However, when evaluated by menopausal status, the benefit was different in post vs. premenopausal women. Study findings indicate that postmenopausal women with 1–3 positive lymph nodes and an RS of 0–25 can safely forgo adjuvant chemotherapy without compromising IDFS. By comparison, premenopausal women with 1–3 positive notes and an RS of 0–25 are likely to benefit from chemotherapy added to standard adjuvant endocrine therapy.

### Comparison trials of prognostic and predictive multigene assays

Several trials evaluated the differences of the tests available in Italy and their possible non-interchangeability.

#### TransATAC trial [30]

A retrospective analysis of 774 biopsy samples from the TransATAC study, performed on post-menopausal women with ER+, HER2− breast cancer and treated for 5 years with tamoxifen or anastrazole, compared 6 tests: the Oncotype DX test, Prosigna, EndoPredict, Breast Cancer Index (BCI), Clinical Treatment Score and immunohistochemistry with 4-markers. The results showed that EndoPredict (EPclin), Prosigna and BCI can better define the prognosis. However, the results of this analysis do not seem to be conclusive, for several reasons:

- All these trials have no predictive value, and the statistical significance has been evaluated with the Chi-square method, useful from a prognostic point of view, but not from a clinical point of view.
- Compared to these trials, EPclin also includes in the evaluation clinical parameters that extend the distance between high and low risk. However, EPclin is particularly useful in attributing a prognostic value to the considered parameters, but not a predictive value of the effectiveness of CT.

**Figure 13:**
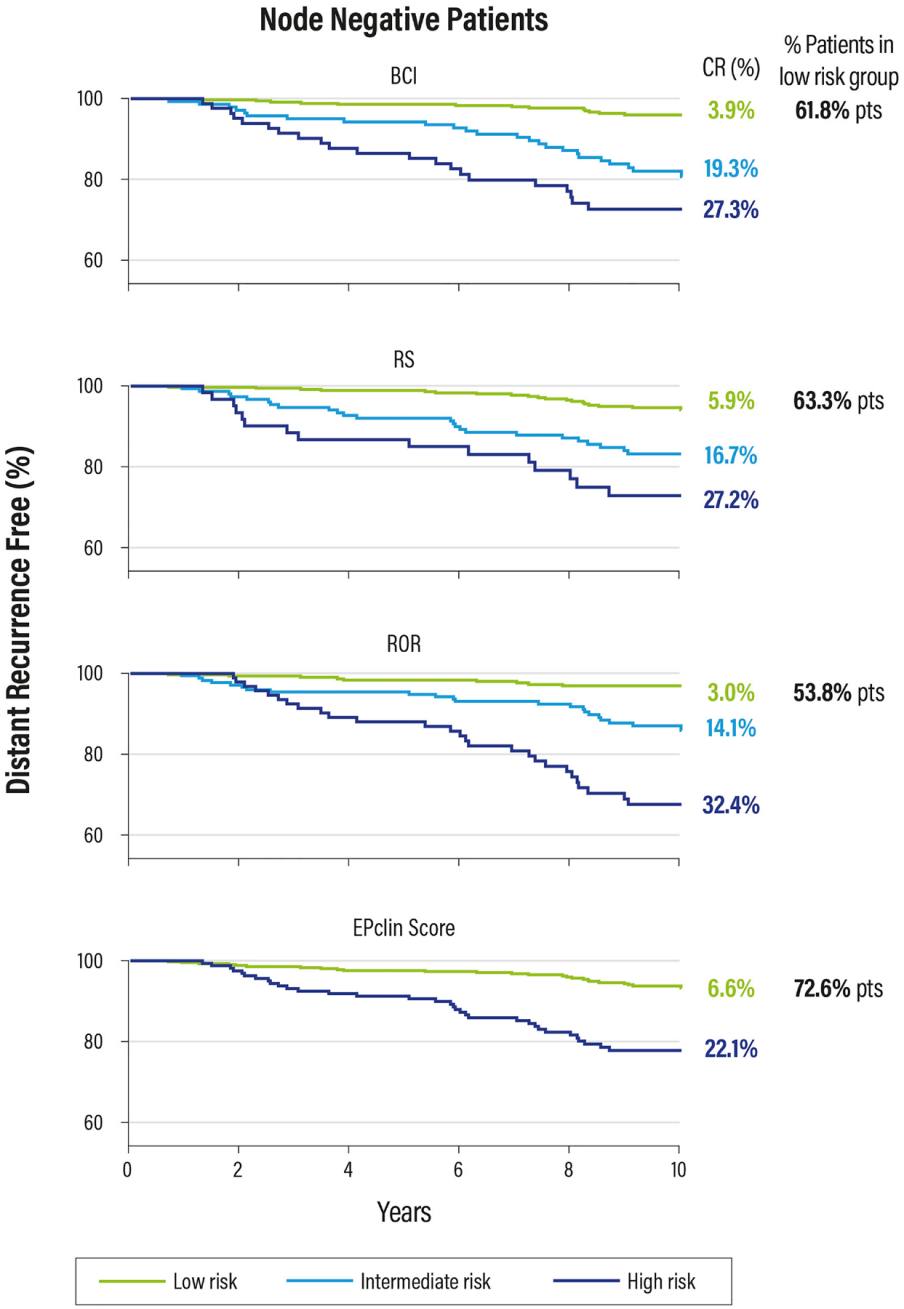
Kaplan Meier plot for relapse risk at 0–10 years in patients with negative nodes.

These data show that these tests can be considered interchangeable from a prognostic point of view, but only the Oncotype DX test is able to predict a benefit form CT (Figure 13).

#### OPTIMA trial [33]

**Figure 14:**
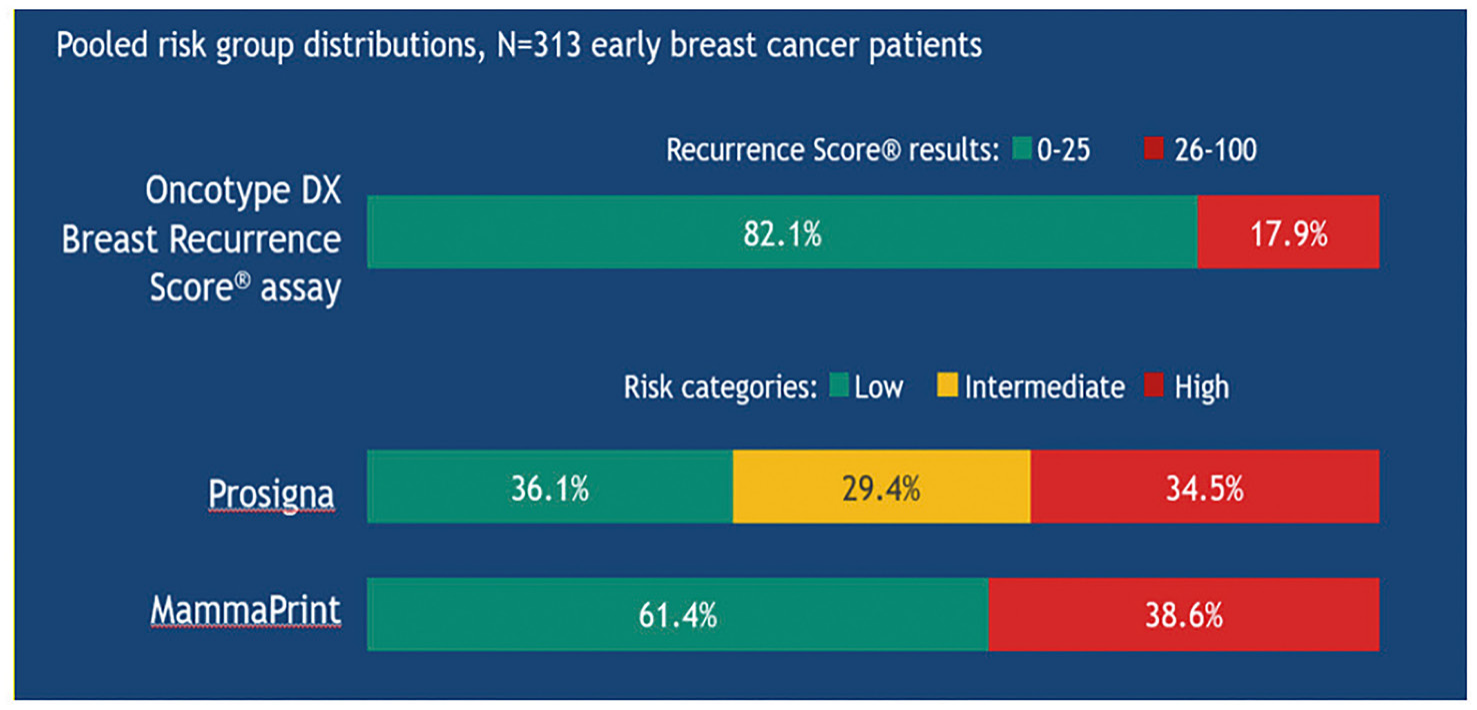
Risk stratification in the OPTIMA trial using different diagnostic tests in the same patients with early breast cancer.

The OPTIMA trial, performed in 35 UK hospitals, evaluated the outcomes of 3 out of 4 genomic tests (the Oncotype DX test, MammaPrint and Prosigna) on patients over 40 years with HR+/HER2− early breast cancer and lymph nodes involvement (1 to 9) or, alternatively, a T >3 cm and N0. The results showed non-homogeneous risk indexes, calculated on the same sample from the same patient (Figure 14).

At individual level, the tests may give different results in terms of both risk and tumour subtype attribution (when predicted by the test).

Today, the Oncotype DX test is the only genomic test able to significantly reduce the number of patients treated with CT (17.9% of patients; RS results 26–100) compared, for example, to MammaPrint^®^ and Prosigna^®^, that target a potential over-treatment (34.5% and 38.6% of patients, respectively) [33, 34].

### Comparison of the clinical utility of the 4 tests approved in Italy

A comparative analysis (38 studies) on the clinical utility of the 4 genomic tests was presented at the ASCO Congress 2018 [35]. The analysis focused on the use of CT in patients with ER+ lymph nodes negative early breast cancer. The results showed that the use of the Oncotype DX test is associated to a lower CT use and lower costs (Table 3).

**Table 3:** Comparative analysis of the effect of genomic tests on chemotherapy

	No test	EndoPredict	MammaPrint	Oncotype DX test	Prosigna
CT use, %	51	56	64	31	49
DR over 10 years, *n*	271	259	269	241	273
ED + H over 10 years, *n*	2260	2274	2435	1630	2113
10-tears total cost of care	$72.9 M	$95.1 M	$102.2 M	$67.5 M	$88.0 M

Abbreviations: CT: Chemotherapy; DR: distant relapse; ED + H: Emergence Department + Hospitalization.

The planned outcomes and costs were calculated on an incidence of 31 women with N0, ER + early breast cancer over 100,000 in 10 years (*n* = 5,574).

## THE DECISION IMPACT OF THE ONCOTYPE DX TEST ON THE CLINICAL PRACTICE

Data from real clinical practice provide additional information to that from randomised controlled trials, allowing for a more realistic situation.

### The surveillance, epidemiology, and end results registry (SEER)

Data from the SEER, which collects cancer incidence data from 1973 to date in the US population, were analyzed in an observational study involving 80,605 patients with HR+, HER2- and negative or positive lymph nodes (up to 3) early breast cancer. The results confirmed that the Oncotype DX test is able to accurately identify patients who may have a good clinical outcome with hormone therapy alone [36]. A statistically significant positive association (*p* < 0.001) was found between the RS results and breast cancer-specific mortality (BCSM) and chemotherapy after normalization by number of positive lymph nodes, age, size and tumor grade. In particular, at the 9 years follow-up, BCSM was less than 4% in N0 patients with RS results 0–25 and in N1 patients with RS results 0–17 who had not received CT; In conclusion, a low RS result in N0 or N1 patients (up to 3 lymph nodes) was able to identify the vast majority, more than 70% of patients who have excellent long-term outcome without CT; on the contrary, high RS results (26–100) can identify the minority of patients who could significantly benefit from adjuvant CT for reducing BCSM. Real-word results from the SEER Registry confirm the high prognostic value of the Oncotype DX test and are supportive of its predictive value for the benefit from CT regardless of the nodal status.

A further analysis of the SEER registry was recently published and is particularly interesting [37] because it shows for the first time that patients undergoing the Oncotype DX test have a better overall survival than patients who were not tested. Although this analysis includes a selection bias, this is the largest and most current clinical practice-based registry evaluating the trend of the Oncotype DX test use in N0 and N1 patients.

### The national cancer database (NCDB)

An interesting analysis in patients with grade 3 tumours enrolled in NCDB, a registry similar to the SEER that collects data from breast cancer patients [38]. These observations confirmed that most patients with grade 3 tumors, despite the high-clinical risk, have low RS results and excellent outcome with hormone therapy alone.

### The clalit registry (CHS)

In 2006, the Israel health care system Clalit (CHS) approved the refunding of the Oncotype DX test for patients with HR+, HER2-, node-negative breast cancer and extended it in 2008 to patients with node-positive (1–3 positive nodes) disease. The data of the Clalit registry were separately analyzed for patients with negative and positive lymph nodes, respectively. The results support the use of the hormone therapy in patients with ER+ HER2- breast cancer, even in patients with positive lymph nodes (up to 3) when RS results are 0–17 [24]. The 10-year follow-up results on 1365 patients were presented in occasion of the 16th St. Gallen International Breast Cancer Conference (Vienna 20–23 March 2019) [25]. 243 patients (17.8%) had RS results 0–10, 853 (62.5%) RS results 11–25 and 269 (19.7%) RS results 26–100. The use of CT was in line with the RS results: 0% in RS 0–10 patients; 9.4% in RS 11–25 patients and 69.9% in RS 26–100 patients. After 10 years, RS results were prognostic, with a clear statistical significance for the period 0–5 years (*p* < 0.001) and a trend for the period >5–10 years (*p* = 0.134). In RS 0–25 patients treated with hormone therapy only, the 10-year results are very favourable and are in line with those of similar studies (TAILORx, WGS-PlanB, SEER Registry, new analysis of NSABP B-20) for which the same cutoff values were set.

### PONDx study

The PONDx programme [39] involved 1,738 patients from 27 Italian centers in 6 regions (Lombardia, Lazio, Campania, Abruzzo, Marche and Emilia Romagna). The objective of the study was to evaluate the impact of the Oncotype DX test on decisions to recommend CT or not in addition to hormone therapy, and to characterize the patients who could benefit from the use of the Oncotype DX test. The results of the survey confirmed that the Oncotype DX test can substantially modify the treatment decisions, showing an overall reduction of CT use by up to 49%. Twelve percent of patients, previously recommended hormone therapy alone, were ultimately given also CT in light of the Recurrence Score results.

Reviewing the results of this PONDx study taking into account the refined cut-off values for the Recurrence Score results established with the publication of the TAILORx trial, the absolute number of patients treated with CT was estimated to be halved (48% to 24%): this would lead to, overall, 75% of the patient population being treated with hormone therapy alone. Such treatment changes could lead to further potential savings for the healthcare system and to an increase of the quality of life of patients.

## GENOMIC TESTS IN THE MAIN GUIDELINES AND HTA BODIES

Due to the value of the clinical evidence supporting the Oncotype DX test as the unique prognostic tool able to predict the benefit associated with CT, this test was considered by the main international guidelines (Table 4) [34]. The AIOM 2019 guidelines describe the use of Prognostic Tumor Molecular Tests (TMMP); however, at the time being, these tests are not included in the Italian Livelli Essenziali di Assistenza (LEA) and therefore they are not funded nationally. Therefore, they are currently used without specific institutional rules, but on the basis of the clinical needs of individual cases and the possibility for patients to directly cover the cost [1] except in Lombardy Region, where the 4 tests are reimbursed if they are used to solve therapeutic questions in complex patients. This significant limitation will be hopefully be solved soon based on the recent document: “Test prognostici multigenici (TPM) per guidare la decisione sulla chemioterapia adiuvante nel trattamento del tumore al seno in stadio precoce”, where AGENAS gives a positive opinion on the clinical utility of the genomic tests.

**Table 4:** Oncotype DX test in therapeutic guidelines and HTA for early breast cancer

Guidelines	Recommendation for Oncotype DX test
IQWiG (Germany) 2020	With the results of TAILORx, only Oncotype DX has sufficient evidence to guide adjuvant chemotherapy decisions in patients with early stage, node-negative, invasive breast cancer
NICE (UK) 2018	Only test considered to predict chemotherapy benefit, therefore providing a cost-effective option in patients with early stage, node-negative and micrometastatic breast cancer
AgeNaS (Italy) 2019	Predictive and prognostic value
St. Gallen (EU) 2019	Test strongly endorsed for guiding adjuvant CT treatment decisions in both node-negative and node-positive early breast cancer. TAILORx cutoffs to guide decisions in node-negative patients
ESMO (EU) 2019	Test may be used to gain additional prognostic and/or predictive information with 1A evidence to complement pathology assessment and to predict the benefit of adjuvant chemotherapy
AJCC (US) 2017	The Oncotype DX Breast Recurrence Score test is the only multigene assay recommended to guide chemotherapy decisions with level IA evidence
ASCO (US) 2019	The Oncotype DX test may be used to guide decisions on adjuvant systemic chemotherapy based on TAILORx cutoffs in node-negative patients with ER-positive, HER2-negative breast cancer
NCCN (US) 2020	Only assay recognized by NCCN Guidelines to predict adjuvant chemotherapy benefit and the only assay classified as the “preferred” multigene test in node-negative patients with HR-positive, HER2-negative breast cancer1
AIOM (Italy) 2019	Test with prospective validation with RCT

Abbreviations: CT: chemotherapy; HER2: epidermal growth factor 2; HR: hormonal receptor; RCT: randomized clinical trial.

All major tests are considered by international guidelines, whose grade of recommendation ranges from strong to moderate depending on the clinical evidence and the type of trials (prospective or retrospective). Recently, the IQWiG (German Institute for Quality and Efficiency in Healthcare) published a review on the evaluation of the benefits of genomic tests: it states that the Oncotype DX test is the only test that able to drive decisions on adjuvant CT in breast cancer.

The latest NCCN (National Comprehensive Cancer Network) guidelines for breast cancer also strongly recommend the Oncotype DX test as the tool able to predict the benefit from an adjuvant CT in patients with both node-negative and positive lymph node breast cancer; the Oncotype DX test is the only genomic test recognized as “Preferred”.

According to AIOM 2019 guidelines, the highest level of evidence (IA) was conferred to the Oncotype DX test; a further confirmation comes from the inclusion of the Oncotype DX test, as the only genomic panel, in the AJCC staging manual, following the prospective level data that support its usefulness.

## CONCLUSIONS

Since many patients with a HR+, HER2- early breast cancer do not benefit from an adjuvant CT, there is a need to have a molecular rationale for identifying patients who can benefit from CT, as well those for whom CT would not add significant clinical benefit.

All multigene assays have a prognostic value, but only the Oncotype DX test has been shown to be able to identify which may benefit from adjuvant CT. Higher values of other genomic tests, such as Prosigna or EndoPredict, although prognostic, are not directly actionable, based on the evidence from clinical trials [13]. In addition, the MINDACT trial (Microarray In Node-Negative and 1 to 3 Positive Lymph Node Disease May Avoid Chemotherapy) demonstrated that MammaPrint has no a predictive value, because it is unable to identify patients who may benefit from CT [14].

Studies comparing different genomic tests showed that they are not interchangeable, because they provide different results: MammaPrint, Prosigna and EndoPredict can identify a higher rate of “high risk” patients than the Oncotype DX test (which defines only a minority of high risk patients), with the risk of an absolutely unjustified over-treatment without any clinical benefit.

To date, the 21-gene assay (Oncotype DX test Breast Recurrence Score) is the only test developed and validated, with a level of evidence 1A, to be *clinically useful*, i.e. able to predict the benefit from an adjuvant CT and therefore suitable to guide the therapeutic choice based on RS results. Clinical evidences from randomised controlled trials show that RS results 26–100 (on 0–100 scale) can predict a significant absolute benefit from the adjuvant CT, while RS results 0–25 indicate a minimal or totally absent benefit [26, 28] The scientific evidence supporting the Oncotype DX test shows that the adjuvant CT can be avoided in most HR+/HER2- patients. More attention should be given to women aged ≤50 years, for whom chemotherapy benefit may be clinically relevant starting with a RS result of 16.
